# On the release of *cppxfel* for processing X-ray free-electron laser images^1^

**DOI:** 10.1107/S1600576716006981

**Published:** 2016-05-11

**Authors:** Helen Mary Ginn, Gwyndaf Evans, Nicholas K. Sauter, David Ian Stuart

**Affiliations:** a Division of Structural Biology, Wellcome Trust Centre for Human Genetics, Roosevelt Drive, Oxford, Oxfordshire OX3 7BN, UK; b Diamond House, Harwell Science and Innovation Campus, Fermi Avenue, Didcot, Oxfordshire OX11 QX, UK; c Molecular Biophysics and Integrated Bioimaging Division, Lawrence Berkeley National Laboratory, 1 Cyclotron Road, Berkeley, California 94720, USA

**Keywords:** X-ray free-electron lasers, XFELS, serial femtosecond crystallography, data analysis, computer programs

## Abstract

As serial femtosecond crystallography expands towards a variety of delivery methods, including chip-based methods, and smaller collected data sets, the requirement to optimize the data analysis to produce maximum structure quality is becoming increasingly pressing. Here *cppxfel*, a software package primarily written in C++, which showcases several data analysis techniques, is released. This software package presently indexes images using DIALS (diffraction integration for advanced light sources) and performs an initial orientation matrix refinement, followed by post-refinement of individual images against a reference data set. *Cppxfel* is released with the hope that the unique and useful elements of this package can be repurposed for existing software packages. However, as released, it produces high-quality crystal structures and is therefore likely to be also useful to experienced users of X-ray free-electron laser (XFEL) software who wish to maximize the information extracted from a limited number of XFEL images.

## Abstract 2

Here *cppxfel*, a software package for integration and post-refinement of serial femtosecond crystallography data, is released.

## 1. Introduction   

X-ray free-electron lasers (XFELs) provide a source of X-ray light in the form of short ∼50 fs pulses, several orders of magnitude brighter than a synchrotron beam (Neutze *et al.*, 2000 ▸; Chapman *et al.*, 2011 ▸). Crystallographic methods can be extended to otherwise intractable micro- or nanocrystalline samples, where the majority of crystal destruction takes place after diffraction has occurred (Chapman *et al.*, 2014 ▸). This paves the way towards reconstructions of electron density from single-molecule diffraction patterns (Ekeberg *et al.*, 2015 ▸). In addition, time-resolved studies on the picosecond or femtosecond timescale are now possible owing to the precision of pulse timing, the very short pulse length and the considerable energy in each pulse (Neutze & Moffat, 2012 ▸). These studies in particular require a very large number of crystals in order to produce enough data per time point to reconstruct a movie of motion, and thus much effort has been devoted to reducing the number of images required to reconstruct high-quality electron density (Hattne *et al.*, 2014 ▸). There are now a number of software packages and their subsequent improvements employing data analysis methods that are capable of processing XFEL data sets, including *CrystFEL* (Kirian *et al.*, 2010 ▸; White *et al.*, 2012 ▸, 2013 ▸) and *cctbx.xfel* (Hattne *et al.*, 2014 ▸; Sauter *et al.*, 2013 ▸, 2014 ▸; Sauter, 2015 ▸). *CrystFEL* has recently benefited from additional algorithms for orientation matrix refinement and modelling partiality (White *et al.*, 2016 ▸).

The applications to soluble proteins include the determination of unknown structures by molecular replacement (notably for a type of cypovirus polyhedrin; Ginn, Messer­schmidt *et al.*, 2015 ▸), proof of principle demonstrations of phasing by use of anomalous scattering (Barends *et al.*, 2013 ▸, 2014 ▸; Nakane *et al.*, 2015 ▸), and time-resolved studies on photoactive yellow protein (Tenboer *et al.*, 2014 ▸) and myo­globin (Barends *et al.*, 2015 ▸). Membrane proteins analysed include photosystem I (Chapman *et al.*, 2011 ▸), photosystem II (Kern *et al.*, 2012 ▸, 2013 ▸, 2014 ▸; Kupitz *et al.*, 2014 ▸; Suga *et al.*, 2015 ▸), rhodopsin bound to arrestin (Kang *et al.*, 2015 ▸) and a human serotonin receptor (Liu *et al.*, 2013 ▸).

Here an open-source software suite (*cppxfel*) is provided to showcase methods which have been previously published (Ginn, Messerschmidt *et al.*, 2015 ▸; Ginn, Brewster *et al.*, 2015 ▸). Implementation of these methods in other software packages is keenly encouraged. The DIALS (diffraction integration for advanced light sources) framework (Waterman *et al.*, 2013 ▸) encompasses the indexing and integration of images initially from synchrotron data using a wide range of optional methods with a clean command line interface. DIALS is also capable of indexing XFEL still images and is currently employed to produce indexing results which can be passed into the *cppxfel* integration and post-refinement pipeline to produce merged reflection lists. A summary of how to use the *cppxfel* software package to process XFEL stills is provided here, along with the tunable parameters of major interest for the user.

## 2. Description of the methods and implementation   

### 2.1. Overview of *cppxfel*-specific features   

*Cppxfel* currently creates merged reflection lists in MTZ format from image files which have been processed by the DIALS indexing algorithms (Waterman *et al.*, 2013 ▸). To improve the ease of use, we include with this *cppxfel* release scripts for indexing still images using DIALS.

There are two major innovations in the strategy employed by *cppxfel* which are likely to be of immediate value to other software: (*a*) the initial orientation matrix refinement as described by Ginn, Messerschmidt *et al.* (2015 ▸), and (*b*) the nature of the partiality model, which has been described by Ginn, Brewster *et al.* (2015 ▸).

The justification for the strategy employed during initial orientation matrix refinement, which has been extended to include refinement of unit-cell lengths, is that over-prediction can reveal spots that are in the correct position but too weak to be picked up by spot-finding algorithms. A small increase in photon counts at any detector pixel is likely to be noise but, if found at a position which is already known to correspond to a nearby Miller index, has a greater probability of being a true signal. By using this information, the number of spots which can be used to refine the orientation matrix is increased substantially, leading to a more accurate estimation of the orientation. As a result, this method is used in *cppxfel* to perform initial refinement of the orientation and unit-cell parameters.

**Figure 1:**
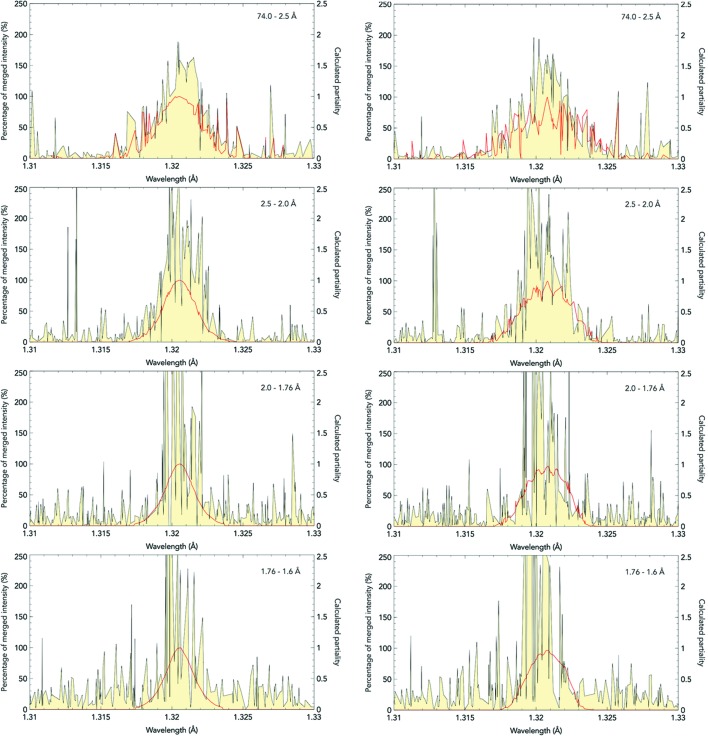
Plotting calculated partiality (red line) against estimate of partiality (yellow fill) by comparing against the reference data set, plotted against the Ewald sphere wavelength for the midpoint of each reflection. These are two models plotted vertically in four resolution shells to 1.6 Å resolution. The left and right models use two different bases: on the left, post-refinement has been carried out with a bandwidth of 0.18% and 0° mosaicity. On the right, the bandwidth was lowered to 0.07% and the mosaicity was increased to 0.03°. Both the bandwidth model and the mosaicity model were calculated with a super-Gaussian exponent of 1.5. The four panels on each side correspond to reflections at increasing resolution. The agreement is fairly close for both models at mid to high resolution, but the mosaicity model has a more erratic structure at low resolution. Overall, the correlation coefficient is reduced for the mosaicity model (89%) compared to the bandwidth model (96%), which suggests that a bandwidth model is preferred for the highly ordered CPV17 polyhedrin crystals from which these data were collected.

The innovative partiality model, which is introduced during post-refinement, combines both bandwidth- and mosaicity-based models. This part of the pipeline fine-tunes the orientation of the unit cell, the wavelength of the XFEL pulse, and the crystal domain size and mosaicity against a reference data set, to achieve high-quality estimations of the full intensities of individual reflections. This model differs from others in the literature in explicitly modelling both bandwidth and mosaicity. The program can use a bandwidth-based model, a mosaicity-based model or a combination of the two. A bandwidth-based model would be best suited to low-mosaicity crystals where reflections are mainly illuminated by the spread of photon wavelengths. However, a mosaicity-based model may be better suited to experimental setups which are fitted with a monochromator. As an example of the differences between bandwidth and mosaicity models, the effects of the two models on a low-mosaicity crystal of CPV17 polyhedrin are shown in Fig. 1 ▸.

### 2.2. Programming environment and implementation   

*Cppxfel* is almost entirely written in C++ but is executed at the highest level using Python. It is designed to be placed within the Computational Crystallography Toolbox (*cctbx*) project (Grosse-Kunstleve *et al.*, 2002 ▸) distribution, and although *cctbx.xfel* is not strictly necessary for compilation, many of the features of *cctbx.xfel* are useful for analysis and it is therefore recommended. *Cppxfel* supports threading within the C++ code on a single machine using the Boost thread library, which is now delivered along with the standard *cctbx* distribution. Machines require a C++ compiler with the c++0x standard available. The code was developed and tested on 64 bit CentOS 6.*x* systems (*i.e.* gcc 4.4.7), DIALS version 1.0-141-g20c8340, *cctbx* build dev-580, to create *cppxfel* version 1.1. The code should be compatible with versions as early as gcc version 4.3 if an earlier version is required for compilation. For the purposes of DIALS analysis, images should be in the same pickle format (Python’s serialization format) used for *cctbx.xfel*. These can be generated directly from the raw streams using a PSDM-enabled *cctbx.xfel* installation, or converted from another supported file format (including *.cbf files which may be generated from synchrotron sources) using the command cxi.image2pickle, also contained within the *cctbx.xfel* distribution. To streamline the implementation of *cppxfel*, the next release will work with the HDF5 image format which is becoming a *de facto* standard for diffraction data (The HDF Group, 1997 ▸), such as that output from the new data processing pipeline at SACLA (Nakane *et al.*, 2016 ▸).

## 3. Optimal use of *cppxfel*   

**Figure 2:**
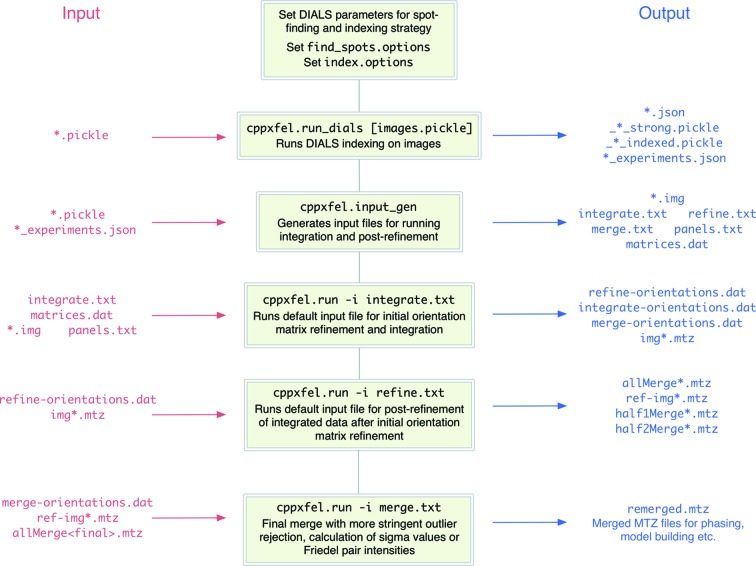
Workflow for executing indexing, initial orientation matrix refinement and post-refinement of individual images.

The software package includes a data set of 1000 Cypovirus polyhedra type 17 (CPV17) diffraction images for which structures have been previously published (Ginn, Messerschmidt *et al.*, 2015 ▸; Ginn, Brewster *et al.*, 2015 ▸) as an example. The data processing is broadly separated into four stages: (i) indexing with DIALS, (ii) initial refinement and integration, (iii) post-refinement, and (iv) final merge (Fig. 2 ▸). This section discusses the optimal use of *cppxfel*, which users may wish to consult for their particular study. The default parameters have been chosen to process the majority of data sets well, but modifying certain parameters can often bring about improvements in the quality of the final data set.

### 3.1. Indexing   

Images are read in and processed singly by DIALS. A Python script is provided to automate this and can be run with  cppxfel.run_dials *.pickle. This will import single images, run spot finding and attempt to index each image. The parameters for these three steps can be customized by changing the option files find_spots.options and index.options which are appended to the command line call within the script. These can be used to change spot-finding parameters or provide target unit cells and space groups for indexing. Refer to the DIALS web site tutorials for more information (http://dials.github.io/documentation/tutorials/index.html). Depending on the data one may find it useful to enable the indexing of multiple crystal lattices (Gildea *et al.*, 2014 ▸).

After individual images have been indexed, the successfully indexed images must be prepared for entry into the *cppxfel* pipeline by running cppxfel.input_gen. This will search for successful images in the current directory and generate prototype input scripts which can be immediately fed into *cppxfel* or altered to improve the refinement strategy. These include the standard scripts integrate.txt, 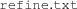 and 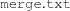.

### 3.2. Initial orientation matrix refinement and integration   

*Cppxfel* is governed by input text files with a simple parameter and value entry format, followed by an optional list of commands. These are supplied to the program by running cppxfel.run -i inputfile.txt. The default input files are 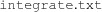, 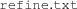 and 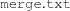. The first carries out initial orientation matrix refinement and integration, generating a single MTZ file per image, the next runs post-refinement on those MTZ files, while the last recalculates sigma values in the final merge.

The main parameters of concern for integration are those defining the background and integration box, any required rough correction of detector geometry (which should not be required if detector geometry has already been corrected), the threshold at which a spot may correspond to a strong reflection, the extent of over-prediction, and parameters concerning the treatment of the unit cell and its refinement.

The parameter METROLOGY_SEARCH_SIZE can be used to roughly correct for detector geometry errors by searching for a nearby maximum pixel value according to the protocol defined previously (Ginn, Messerschmidt *et al.*, 2015 ▸). For example, a search size of 5 will recentre the integration box on the pixel with the maximum value up to 5 pixels away from the predicted spot position.

**Figure 3:**
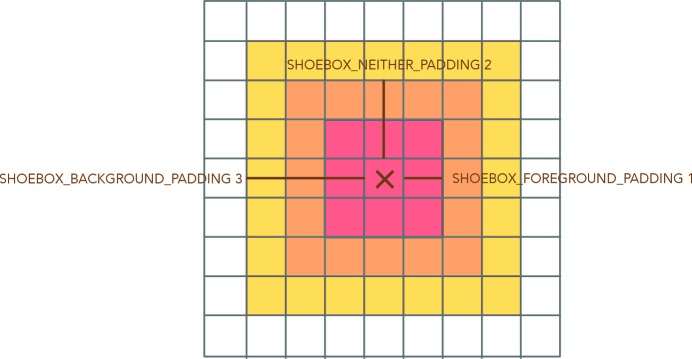
Simple integration box available in *cppxfel*, defined by the three parameters shown specifying the size of the boxes of foreground, neither and background flags.

The simple integration box is primarily governed by three parameters: SHOEBOX_FOREGROUND_PADDING, SHOEBOX_NEITHER_PADDING and SHOEBOX_BACKGROUND_PADDING. These parameters define squares of integer distances from the central pixel, where pixels are treated as foreground signal, ignored pixels or background pixels as in Fig. 3 ▸. This is a very simple treatment of spot integration, but it has been found to be capable of generating high-quality structures. Spots are integrated using a simple summation of foreground pixels and removal of the estimation of background in this area. Integrated positions are considered to be potential reflections for the purposes of initial orientation matrix refinement if the measure of their signal exceeds a certain value defined by INTENSITY_THRESHOLD. The toggle ABSOLUTE_INTENSITY, which can be set to 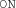 or 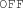, determines whether this uses the raw intensity value or the 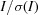 value.

The method used by initial orientation matrix refinement and the subsequent post-refinement relies on over-prediction of illuminated Miller indices. Some crystals may have a higher mosaicity than others, or have a poorer starting orientation matrix, and thus require a wider prediction of reflections. These are governed by two parameters. OVER_PRED_BANDWIDTH primarily increases over-prediction at high resolution by assuming a very wide bandwidth of the beam energy, and may need expanding from the default of 3% (representing the spread of two standard deviations of a super-Gaussian model for beam energy). In contrast OVER_PRED_RLP_SIZE increases over-prediction at low resolution by expanding the size of the reciprocal lattice points, thus increasing their likelihood of intersecting the illuminated Ewald spheres. OVER_PRED_RLP_SIZE may need to be increased from the default of 2 × 10^−4^ Å^−1^ radius (corresponding to a crystal domain size of 500 nm) if there are not enough predicted reflections at low resolution evident in later processing.

Finally, unit-cell dimensions from indexing can vary beyond the variation expected for a biological sample. This is due to uncertainty in other parameters and also because each image is treated independently and, to a first approximation, samples a central section of the reciprocal lattice which is often insufficient to properly define all unit-cell dimensions. Thus, there is often a benefit in resetting the unit-cell dimensions to their expected values, if they are known. This is governed by the toggle parameter FIX_UNIT_CELL, which, if set to ON, will reset the unit cell to the six parameters defined in UNIT_CELL. The unit-cell dimensions of cubic space groups are perfectly correlated with the beam energy, and hence further refinement is not needed. However, other space groups may require refinement of unit-cell dimensions. The unit-cell lengths can be refined by toggling the parameters REFINE_UNIT_CELL_A, REFINE_UNIT_CELL_B and REFINE_UNIT_CELL_C, and the unit-cell angles by REFINE_UNIT_CELL_ALPHA, REFINE_UNIT_CELL_BETA and REFINE_UNIT_CELL_GAMMA. *Cppxfel* ensures that unit-cell lengths are kept identical if strictly required by the chosen space group.

**Figure 4:**
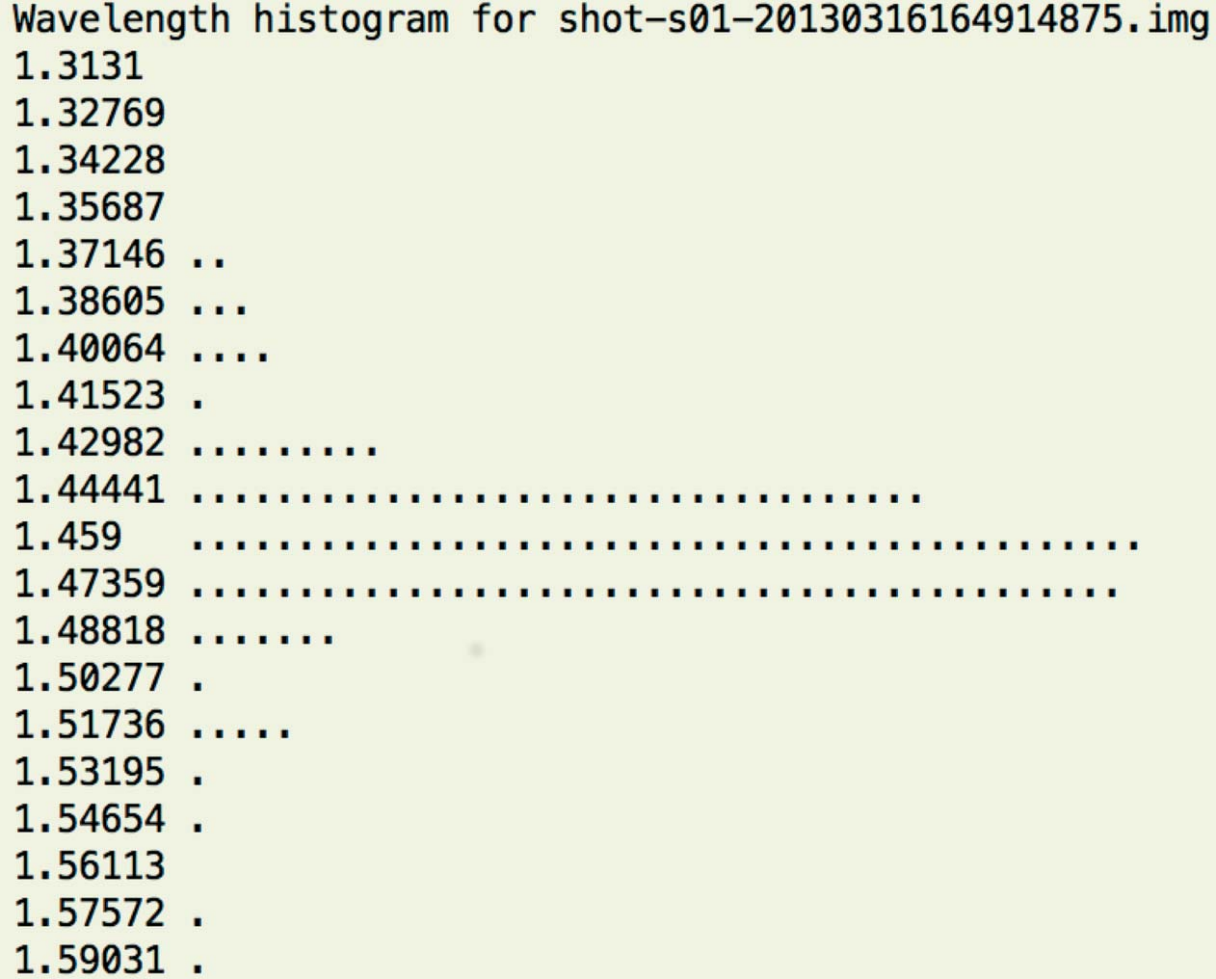
Example of an Ewald sphere wavelength histogram produced during initial orientation matrix refinement, which is indicative of a successfully refined image.

The output from integration will produce histograms showing the distribution of strong reflections over the corresponding Ewald sphere wavelengths at which they are illuminated (Fig. 4 ▸). This is primarily governed by the crystal orientation, and optionally by altering the unit-cell dimensions as well. One should modify these parameters to aim to produce histograms which are within a narrow range of Ewald sphere wavelengths with as few reflections as possible at the edges of the distribution. The results of this integration stage are a series of MTZ files, one per crystal. If DIALS was configured to index multiple crystals per lattice, this may result in more MTZ files than image files. The software also produces a list of updated matrices to send into the next stage of the pipeline, post-refinement. These are stored in a plain text file as a default set of files *-orientations.dat which can be fed into the various stages of data processing.

### 3.3. Post-refinement   

Post-refinement is implemented as previously described (Ginn, Brewster *et al.*, 2015 ▸), with some improvements and alterations. There are certain parameters to carefully consider changing for optimal post-refinement. The most important parameters govern the choice of starting values for refining models for individual images and their step sizes during minimization. The following parameters in particular may require changing from their default values: (*a*) the starting value of the reciprocal lattice point size, (*b*) the parameters in the calculation of mean wavelength and (*c*) the step size of orientation matrix refinement. The reciprocal lattice point size determines the radius of the reciprocal lattice point in reciprocal ångströms at the origin of reciprocal space, before any additions due to mosaicity. The mean wavelength is the estimated wavelength of the X-ray pulse for that particular exposure, bearing in mind this scales directly with the overall unit-cell size.

Generally, the default parameters for post-refinement will provide a good quality set of intensities for good quality diffraction obtained at an XFEL. However, the user may wish to tweak parameters to achieve small improvements in the 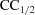 or 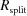 values. For example, the step size of the orientation matrix refinement (STEP_SIZE_ORIENTATION) can specify how widely sampled the function landscape should be in the horizontal and vertical rotation parameters, and although this has a default setting of 0.06°, it may be increased or reduced in order to step over or avoid local minima in the function landscape. It may need to be reduced so as not to leave the over-predicted reflection zone, which would lead to extremely low numbers of reflections being accepted for a number of images. Conversely, it may need increasing in order to sufficiently explore the function landscape to find an appropriate minimum. It is also possible to further refine the unit-cell dimensions. The closer the initial estimate to the correct solution, the more likely it is that an image will refine to the global minimum and not a local minimum. The beginning of post-refinement is also the point at which any indexing ambiguity is broken using an implementation of a cluster algorithm (Brehm & Diederichs, 2014 ▸), which is currently only supported for twofold indexing ambiguities.

Reciprocal lattice point sizes are proportional to the domain size of the crystal and expand with resolution according to mosaicity (Rossmann *et al.*, 1979 ▸). This model is also implemented in *cctbx.xfel* (Sauter *et al.*, 2014 ▸). The starting values of these parameters are defined by INITIAL_RLP_SIZE (default 1 × 10^−4^ Å^−1^, which usually needs to be raised) and INITIAL_MOSAICITY (default 0). Changing the reciprocal lattice point size has the greatest effect at low resolution. Thus, errors in the reciprocal lattice point size can be judged by considering the merging statistics at low resolution and examining plots of partiality agreement by resolution (see §3.5). This can also help judge the requirement for including a mosaicity term: if there is under-prediction at high resolution which is not adequately addressed by the bandwidth parameter.

The illuminated wavelength is calculated by taking the mean Ewald sphere wavelength of all strong reflections. Thus, the threshold of a strong reflection should be set appropriately, using REFINEMENT_INTENSITY_THRESHOLD and specifying an absolute intensity cutoff above which reflections are included in the calculation. If outliers are causing problems in the determination of the beam wavelength, they can be excluded by specifying a wavelength beyond which reflections are excluded. This is determined by lower and upper wavelength boundaries following the keyword WAVELENGTH_RANGE. These parameters may need altering to produce the best merging statistics or can be judged by examining plots of partiality agreement by resolution (see §3.5).

Refinement of unit-cell parameters can be switched on and off by toggling the parameters OPTIMISING_UNIT_CELL_A, OPTIMISING_UNIT_CELL_B and OPTIMISING_UNIT_CELL_C, which can provide further improvements on the initial estimates from the integration stage for appropriate space groups.

Nelder–Mead minimization (Nelder & Mead, 1965 ▸) is used to refine to the local minimum, and the initial starting values for Nelder–Mead can have a great influence on its ability to fully minimize. Hence, the step sizes can be specified for each of the parameters according to the STEP_SIZE_* parameters as defined in the manual.

The primary parameter pertaining to partiality is PARTIALITY_CUTOFF, which determines the minimum partiality which would be accepted for merging and evaluation of the target function per image. The default is set to 0.2, but this may be raised to around 0.3 for poor crystals or lowered to 0.05 for well behaved crystals. The slicing of the numeric integration for partiality calculation, particularly at low resolution where the nest of Ewald spheres (representing different incident energies) is very tightly packed, may need to be finer for reciprocal lattice points of a larger size. The maximum partiality slicing is defined by MAX_SLICES, set to a default of 100, which is employed at resolutions below CAREFUL_RESOLUTION, where care must be taken to ensure adequate numerical integration. If the multiplicity of low-resolution reflections is particularly low, this parameter may need to be increased, as the multiplicity may be due to insufficient slicing to properly estimate how the bandwidth of the beam intersects the reciprocal lattice point. If MAX_SLICES is set too coarse this may result in suspiciously low 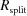 merging statistics and high 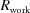 and 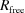 values.

Two methods of outlier rejection are employed, but depending on detector and data quality, one or both may need disabling or modifying. Rejection of reflections on individual images as described previously (Ginn, Brewster *et al.*, 2015 ▸) can be turned off using the parameter CORRELATION_REJECTION. Rejection at the merging stage by standard deviation from the mean can be turned off entirely using the parameter OUTLIER_REJECTION, or the standard deviation threshold can be modified from the default value of 1.8 using OUTLIER_REJECTION_SIGMA.

If a set of reference intensities is available, this can be provided as a path to an MTZ file using the command INITIAL_MTZ. This is used to provide an initial reference set of intensities, which are refined against but not included in the merges at the end of the macrocycles. This allows data sets to be processed with a more than twofold indexing ambiguity if the reference set provided is untwinned.

### 3.4. Final merge   

Running cppxfel.input_gen creates a template input file 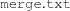 which should be run after 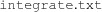 and 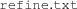. This merging stage can have outlier rejection or image rejection parameters different from earlier stages of refinement. This will create a final MTZ file named 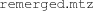 which will have corrected sigma values for use in other crystallographic programs. These are calculated by a weighted standard deviation function divided by the square root of the number of observations, so are more akin to a term of precision. Setting the toggle MERGE_ANOMALOUS will output anomalous differences in Friedel pairs to anomalous_diff.mtz which can be used to assess anomalous data quality.

### 3.5. Analysing the data output   

Merging statistics are a simple measure of data quality for a set of reflections and both 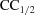 (Karplus & Diederichs, 2012 ▸) and 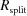 (White *et al.*, 2013 ▸) are output during each macrocycle of post-refinement. Errors in integration or data refinement strategies are likely if these values fail to tend towards 1.0 and 0.0, respectively. One should ensure wavelength histograms as in Fig. 4 ▸ are being obtained during the integration step, and check that the plots of partiality agreement over resolution shells do not show global errors in reciprocal lattice point size or calculation of mean wavelength. Data would be expected to decay with resolution.

Merged MTZ files are written out to files named 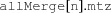 where [n] is the cycle number of post-refinement. Individual halves of the data set used for 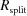 and 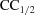 calculation, including a full reflection list, are written to 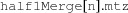 and 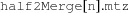 and can be compared after refinement is complete using cppxfel.run -cc half1Merge[n].mtz half2Merge[n].mtz.

Other merging statistics are available within the program for unmerged MTZ files. A large MTZ file containing unmerged data is written to 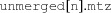 which can be used to calculate 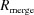, 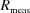 and 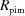 using the command cppxfel.run -command unmerged[n].mtz, where 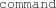 is replaced by 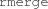, 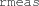 or 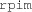.

**Figure 5:**
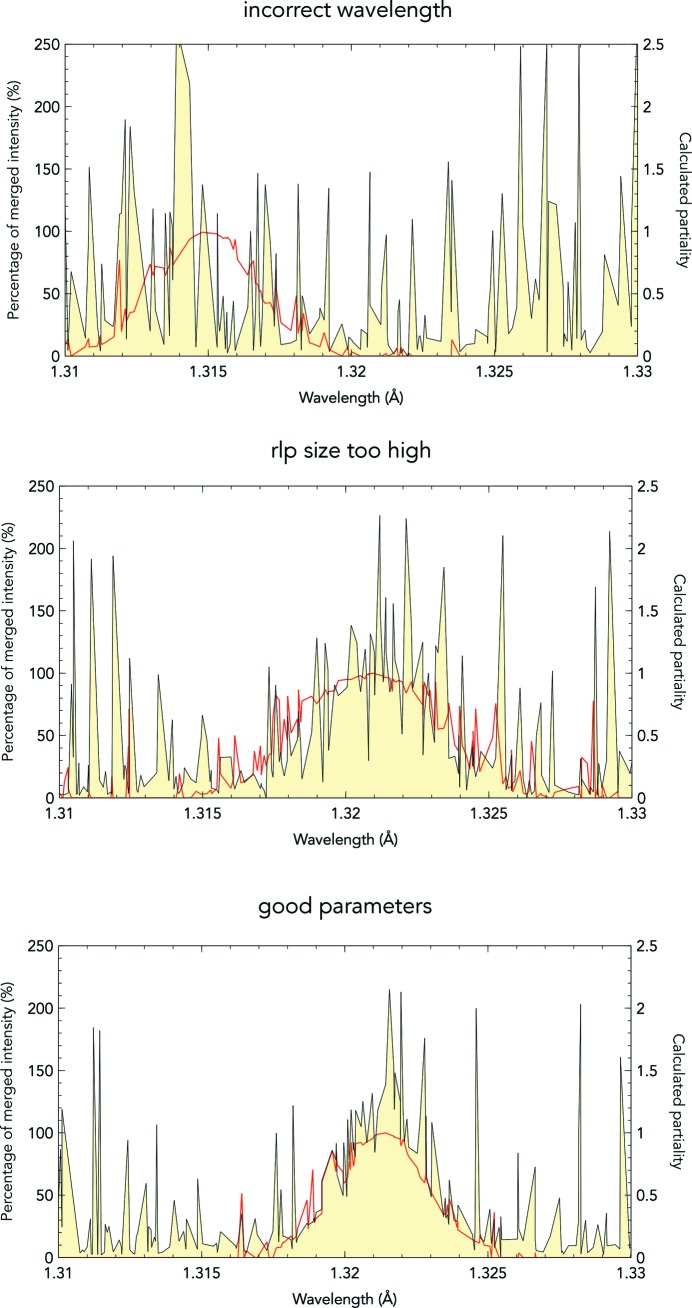
Plotting calculated partiality (red line) against estimate of partiality (yellow fill) by comparing against the reference data set, plotted against the Ewald sphere wavelength for the midpoint of every reflection under 2.5 Å resolution. These plots represent incorrect parameter choices. The mean wavelength of the X-ray pulse was manually reconfigured to be off by 0.005 Å and the effect of this error is shown in the top panel. The crystal is rotated by the minimization method far off the true solution in order to compensate for the incorrect wavelength, to the point that the true wavelength peak is no longer recognizable. The middle panel had the reciprocal lattice point (rlp) size inflated by 100%, also leading to an incorrect crystal rotation to compensate by broadening the distribution of wavelengths. Finally, the bottom panel shows a set of good parameter choices which lead to a good refinement solution. This image come from a data set collected on CPV17 crystals.

It may be useful to analyse the quality of post-refinement of individual crystals against the reference data set to gain the best insight into errors in the post-refinement strategy. To do this, partiality agreement data in CSV format can be generated. This uses the command cppxfel.run -partiality reference.mtz crystal.mtz [maxRes], where 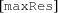 is the maximum resolution for analysis. This will generate CSV files for each of four resolution bins. Each file will contain a list of Ewald sphere wavelengths for each reflection in the crystal MTZ file, the percentage of the merged reflection intensity per reflection, the calculated partiality and the resolution of each reflection. The CSV files will be named partiality_[m].csv, where [m] is the bin number. These can then be imported into any graph drawing software to plot the data and identify pathologies as shown in Fig. 5 ▸.

## 4. Conclusion   

This paper provides an introduction to data processing using *cppxfel* to allow developers to understand the impact of the models used in this software package and to provide a guide to facilitate the effective use of the software by experienced XFEL users. The code is open source, and the associated Wiki entries may be accessed at http://viper.lbl.gov/cctbx.xfel/index.php/Cppxfel. This includes installation instructions and a step-by-step tutorial to process the provided CPV17 polyhedrin diffraction images.
